# Murine Norovirus Interaction with Enterobacter cloacae Leads to Changes in Membrane Stability and Packaging of Lipid and Metabolite Vesicle Content

**DOI:** 10.1128/spectrum.04691-22

**Published:** 2023-03-21

**Authors:** Chanel A. Mosby, Mariola J. Edelmann, Melissa K. Jones

**Affiliations:** a Microbiology and Cell Science Department, IFAS, University of Florida, Gainesville, Florida, USA; The Ohio State University Division of Biosciences

**Keywords:** murine norovirus, commensal bacteria, outer membrane vesicle, lipidomics, metabolomics, microbiome, bacterial extracellular vesicle, *Enterobacter cloacae*, sphingolipids, vesicle content, OMV, bacterial vesicle content, gut microbiome, vesicle biogenesis

## Abstract

Outer membrane vesicles (OMVs) are a primary means of communication for Gram-negative bacteria. The specific role of vesicle components in cellular communication and how components are packaged are still under investigation, but a correlation exists between OMV biogenesis and content. The two primary mechanisms of OMV biogenesis are membrane blebbing and explosive cell lysis, and vesicle content is based on the biogenesis mechanism. Hypervesiculation, which can be induced by stress conditions, also influences OMV content. Norovirus interaction with Enterobacter cloacae induces stress responses leading to increased OMV production and changes in DNA content, protein content, and vesicle size. The presence of genomic DNA and cytoplasmic proteins in these OMVs suggests some of the vesicles are formed by explosive cell lysis, so reduction or loss of these components indicates a shift away from this mechanism of biogenesis. Based on this, further investigation into bacterial stability and OMV content was conducted. Results showed that norovirus induced a dramatic shift in OMV lipid content. Specifically, the increased accumulation of phospholipids is associated with increased blebbing, thereby supporting previous observations that noroviruses shift the mechanism of OMV biogenesis. Slight differences in OMV metabolite content were also observed. While norovirus induced changes in OMV content, it did not change the lipid content of the bacterial outer membrane or the metabolite content of the bacterial cell. Overall, these results indicate that norovirus induces significant changes to OMV lipid architecture and cargo, which may be linked to a change in the mechanism of vesicle biogenesis.

**IMPORTANCE** Extracellular vesicles from commensal bacteria are recognized for their importance in modulating host immune responses, and vesicle content is related to their impact on the host. Therefore, understanding how vesicles are formed and how their content shifts in response to stress conditions is necessary for elucidating their downstream functions. Our recent work has demonstrated that interactions between noroviruses and Enterobacter cloacae induce bacterial stress responses leading to hypervesiculation. In this article, we characterize and compare the lipid and metabolomic cargo of E. cloacae vesicles generated in the presence and absence of norovirus and show that viral interactions induce significant changes in vesicle content. Furthermore, we probe how these changes and changes to the bacterial cell may be indicative of a shift in the mechanism of vesicle biogenesis. Importantly, we find that noroviruses induce significant changes in vesicle lipid architecture and cargo that may be responsible for the immunogenic activity of these vesicles.

## INTRODUCTION

Bacteria secrete extracellular vesicles that range in size between 50 and 250 nm in diameter and are enclosed by the outer membrane (OM) for Gram-negative bacteria or the cytoplasmic membrane for Gram-positive bacteria. These vesicles contain membrane-embedded proteins from their parental bacteria along with periplasmic or cytoplasmic components, depending on the pathway of vesicle biogenesis (1, 2). Outer membrane vesicles (OMVs) serve various functions, including mediation of host–pathogen interactions, interspecies communications in biofilms, and protection for the bacterial cell as a stress response (3 to 6). The mechanism by which these vesicles are formed is still being researched; however, it has been shown that damage to the peptidoglycan cell wall results in vesicle formation (1, 2, 4, 7). For Gram-negative bacteria, there are two proposed routes of membrane vesicle formation: (i) budding off the outer membrane (blebbing) to create OMVs, and (ii) explosive cell lysis leading to the self-assembly of membrane fragments to create outer-inner membrane vesicles (OIMV) and explosive outer-membrane vesicles (EOMV) (2). The different formation routes also lead to differences in vesicle content. Membrane blebbing results in OMVs that consist mainly of the outer membrane and periplasmic lipids, proteins, and lipopolysaccharides (1, 4). OIMVs and EOMVs formed via explosive cell lysis also consist of outer membrane lipids and proteins but have increased amounts of periplasmic proteins, proteins and lipids of the inner membrane, and cytoplasmic material, including nucleic acids (2, 7).

Both commensal and pathogenic bacteria produce OMVs as part of their normal growth conditions, but that production can be affected by external stressors such as temperature, oxygen, envelope stresses, antibiotic treatment, virus exposure, or defects in cell wall remodeling (8 to 13). Stress conditions can lead to hypervesiculation for some bacterial species (5, 9 to 12), and those same stressors also affect the cargo content of the OMVs. For example, Escherichia coli OMVs collected during growth in minimal media showed an altered proteome (14). Stress conditions are not the only drivers of vesicle production changes. Factors such as growth stage can also effect vesicle size and cargo composition as seen in changes to the protein profiles of Helicobacter pylori vesicles at 16, 48, and 72 h of growth (15). Preferential packaging of vesicle content is also an emerging area of research. Urashima et al. showed that an increase in the expression of *ompT* by an enterohemorrhagic E. coli led to increased OmpT in the OMVs, but not in the bacterium itself, supporting the idea of preferential content packaging of the vesicles (16). However, the certainty of selective packaging requires confirmation that the vesicles are budding from the outer membrane and that no contaminates are present.

While not previously thought to be a stressor of commensal bacteria, it was recently shown that E. cloacae interaction with noroviruses, which are eukaryotic enteric viral pathogens, leads to the induction of stress response genes (11). Eukaryotic enteric viruses do not infect bacteria, but they have been shown to bind to the surface of commensal bacteria (17 to 19). E. cloacae, which is a commensal bacterium that can be an opportunistic pathogen, was originally used to study the effects of norovirus and bacterial interactions due to the ability of human norovirus to heavily bind this bacterium in human stool (19 to 21). While much about norovirus pathogenesis is unknown, one well-established characteristic is that noroviruses interact with human histo-blood group antigens (HBGAs) present on the surface of certain cells. E. cloacae has been shown to express HBGA-like molecules, and it is this HBGA-like molecule expression that likely facilitates human norovirus binding (17, 19). Furthermore, E. cloacae has also been associated with enhancement of human norovirus and murine norovirus infections (20, 21). Given the ability of commensal OMVs to influence host immune responses and alter viral infection (3, 22 to 26), characterizing changes in content of this bacterium linked to viral infection was of great interest.

Nanoparticle tracking analysis (NTA) of membrane vesicles produced by Gram-positive and Gram-negative commensal bacteria found that cultures exposed to human or murine noroviruses produced significantly larger amounts of vesicles compared to control cultures, indicating that the stress induced by noroviruses leads to hypervesiculation, as has been shown for other types of bacterial stress (11). Interestingly, interaction with noroviruses also resulted in a reduction in membrane vesicle size and changes in protein and DNA content (11). It has been reported that E. cloacae vesicles contain both cytoplasmic proteins and genomic DNA, suggesting that this bacterium is capable of forming vesicles through explosive cell lysis when grown *in vitro* (27). However, when vesicles are produced in the presence of norovirus, cytoplasmic protein and DNA content decrease, indicating a shift toward another mechanism of vesicle biogenesis, potential membrane blebbing (11). Changes in vesicle size are linked to changes in the lipid profile or protein content of the vesicles, which can be heavily influenced by the vesicle biogenesis route (12, 28). Together, these results indicated that interaction with noroviruses selectively alters membrane vesicle biogenesis. Consequently, the present study further explores this hypothesis by evaluating the impact of murine norovirus (MNV) interaction with E. cloacae on changes to the lipidic and metabolic component of membrane vesicles as well as the cellular stability of these bacteria.

## RESULTS

### Incubation with MNV decreases amount of E. cloacae with membrane damage.

**FIG 1:**
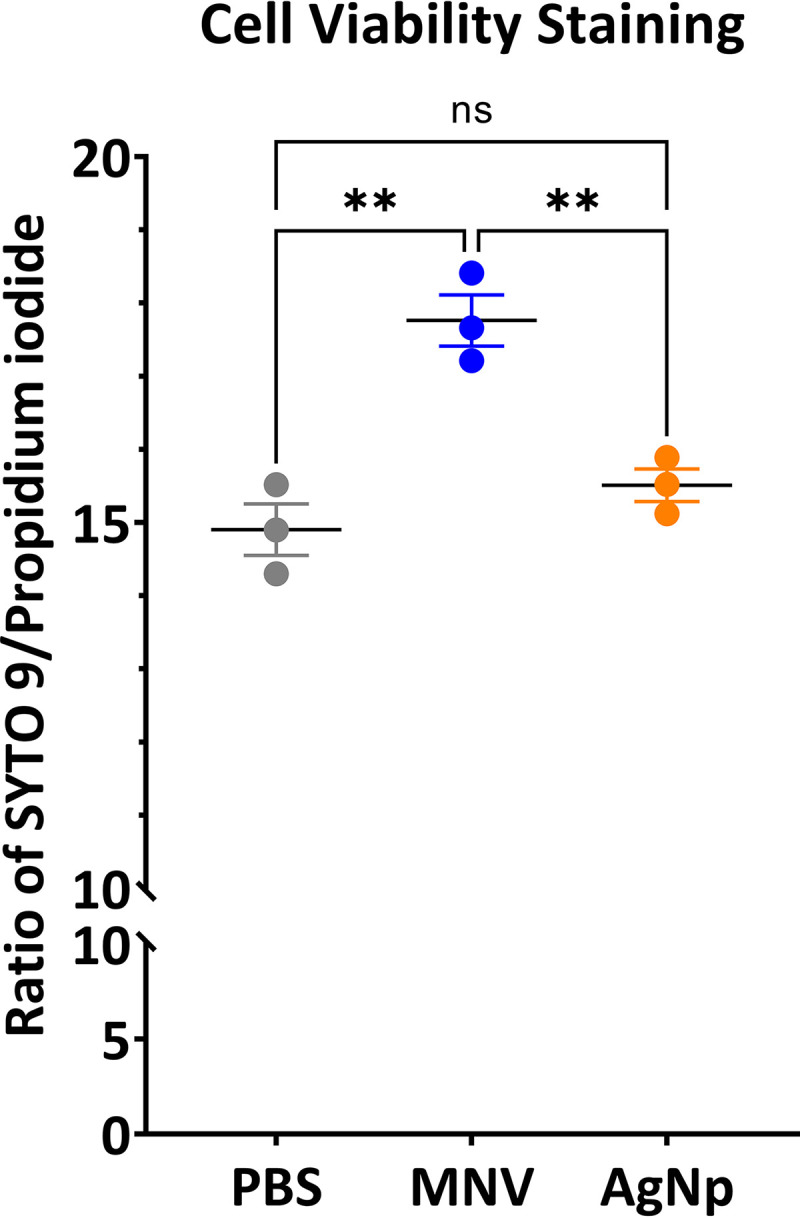
Cell viability results show the ratio of SYTO 9 to propidium iodide for the different conditions in a microplate assay. A higher ratio corresponds to fewer cells with damaged membranes. The statistical significance was determined via a one-way ANOVA with Tukey’s multiple-comparison test. **, *P* < 0.01.

The effect of MNV on the viability of E. cloacae was tested where the number of cells with damaged membranes was evaluated after treatment with MNV or 45-nm silver nanoparticle (AgNp) control compared to phosphate-buffered saline (PBS) control. As with our previous work, PBS and AgNp were used as controls, where AgNp served as an abiotic control of similar physical size to noroviruses but lacking viral antigens, and PBS was the diluent for both MNV and AgNp^12^. A bacterial viability assay using SYTO 9 green-fluorescent stain and propidium iodine red-fluorescent stain was used. SYTO 9 enters cells regardless of membrane integrity, while propidium iodine will enter bacteria with compromised membranes. Bacterial viability assays can be used with fluorescence microscopy and microplate readers. The assay was conducted using the microplate reader to reduce unintentional user bias that may occur with the microscopy method, which requires manual selection of fields of view. Cultures of E. cloacae were incubated for 12 h with either PBS, MNV, or AgNp. The ratio between the SYTO9 fluorescent and the propidium iodide fluorescence was calculated (Fig. 1), where a higher ratio indicates fewer cells with damaged membranes. The AgNp treatments did not lead to significantly different viability from PBS control treatment, but the MNV condition shows a statistically significant difference from the controls, with the ratio indicating fewer cells with damaged membranes in the MNV condition (Fig. 1).

### Murine norovirus interaction results in distinct lipidome of bacterial extracellular vesicles.

Lipidomic analysis was performed on OMVs derived from four biological replicates of E. cloacae incubated with either PBS, 45-nm AgNp, or MNV. Lipid extracts were run on a Thermo Q-Exactive Orbitrap mass spectrometer, and data from the positive and negative ion modes were separately analyzed with LipidMatch software. A combined total of 292 features were identified from the untargeted lipidomics on the positive and negative ion modes across all sample types.

**FIG 2:**
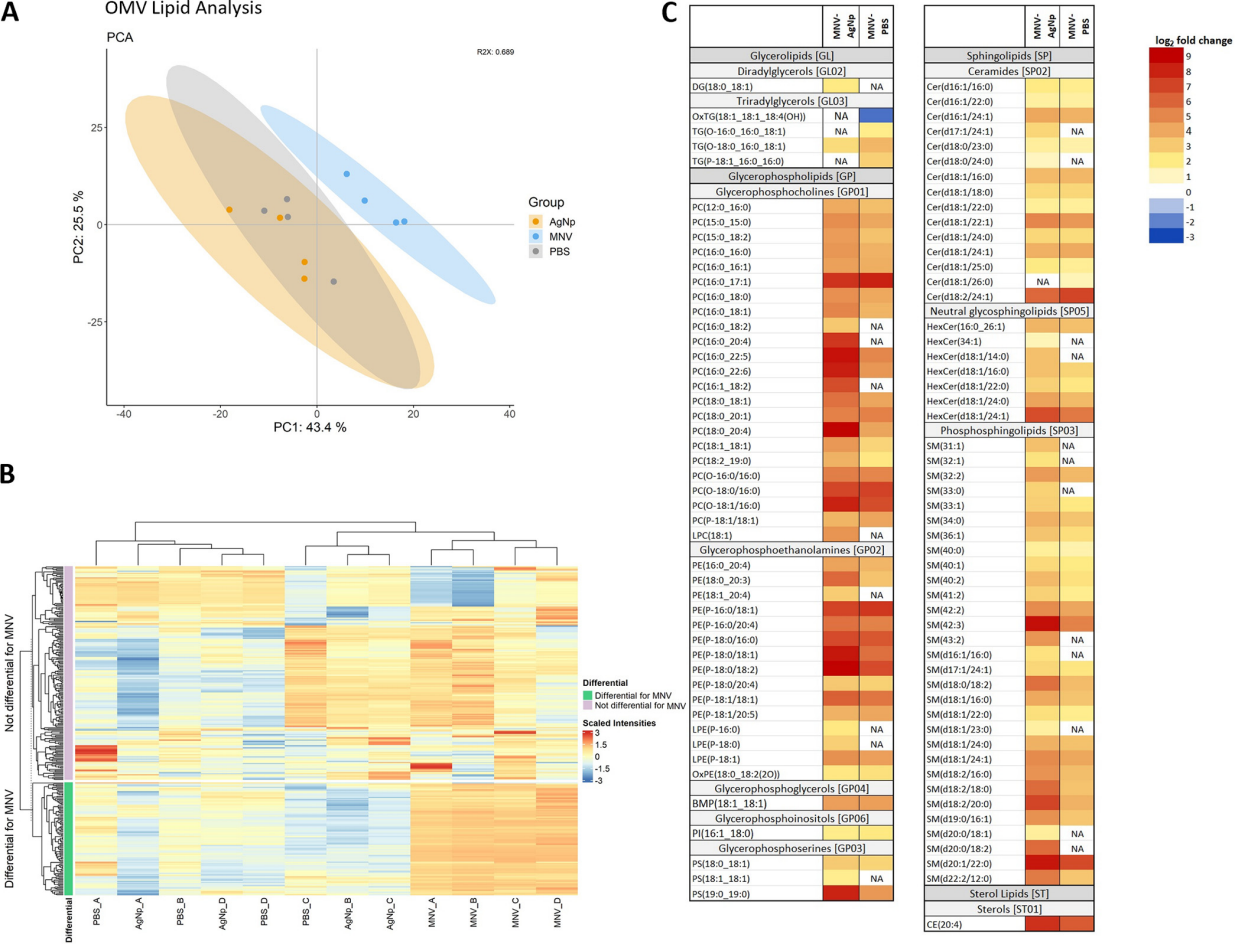
Lipidomic analysis of bEVs produced by Enterobacter cloacae. (A) Principal-component analysis (PCA) plot showing the global difference in lipid composition of E. cloacae bEVs under MNV, AgNp, and PBS conditions. Cultures of E. cloacae were incubated with MNV-1, PBS, or AgNp with PBS and AgNp serving as control conditions. The PBS condition samples are gray, MNV is in blue, and AgNp is in orange. *n* = 4. (B) Heatmap showing the normalized and clr-transformed lipidomics results. The *y* axis coloration highlights lipids found to be differentially abundant in the MNV condition in green, while the lilac coloration shows the lipids not found to be statistically different between the conditions. (C) Heatmap showing lipids that are differentially expressed between MNV and the AgNp and PBS control conditions. Color gradient represents the log_2_-fold change values of the statistically significant lipids, all of which had a false-discovery rate (FDR) of <0.05. NA indicates that particular comparison was not statistically significant.

For a global overview of the differences and similarities between the groups, an unsupervised multivariate analysis was performed using principal-component analysis (PCA) (Fig. 2A). In the PCA plot, the two control groups, PBS and AgNp, overlap each other closely while the MNV group is separate, indicating that the lipid profile of OMVs generated in the presence of the virus is distinct from vesicles produced by the bacterial control groups. Further examination of the overall distribution of the identified lipid species using a heatmap shows a distinct clustering of the MNV samples separate from the PBS and AgNp samples (Fig. 2B). The R package, LipidR, was used to test for differentially abundant lipids using a log_2_-fold change cutoff of ±1 and an adjusted *P* value cutoff of <0.05 (Fig. 2C, Table S1 in the supplemental material) (29). This analysis identified 97 lipids differentially abundant in OMVs generated in the presence of MNV versus in the presence of AgNp and 80 lipids differentially abundant in the presence of MNV versus PBS control. No lipids were seen to be differentially abundant when comparing AgNp versus PBS treatments. Of the identified lipids that met the log_2_-fold change and adjusted *P* value cutoff, only one lipid was seen to have a statistically significant decreased abundance in the MNV versus PBS condition, OxTG(18:1_18:1_18:4(OH)). Of these, 76 lipids were found to be shared, meaning that these lipids were shown to be differentially abundant, with more seen in the MNV condition than in both the PBS and AgNp conditions. Collectively, these results indicate that the lipid content of the bacterial vesicles produced in the presence of MNV is unique compared to the vesicles produced by control cultures, which is consistent with previous work demonstrating that MNV induces changes in E. cloacae vesicle protein and DNA content (11). It is worth noting that these changes may also be related to the MNV-exposed cultures having a different ratio of OMVs to OIMVs compared to the control cultures; however, further vesicle analysis is needed to quantify the vesicle types and determine if any changes in vesicle type ratio occur after MNV exposure.

**FIG 3:**
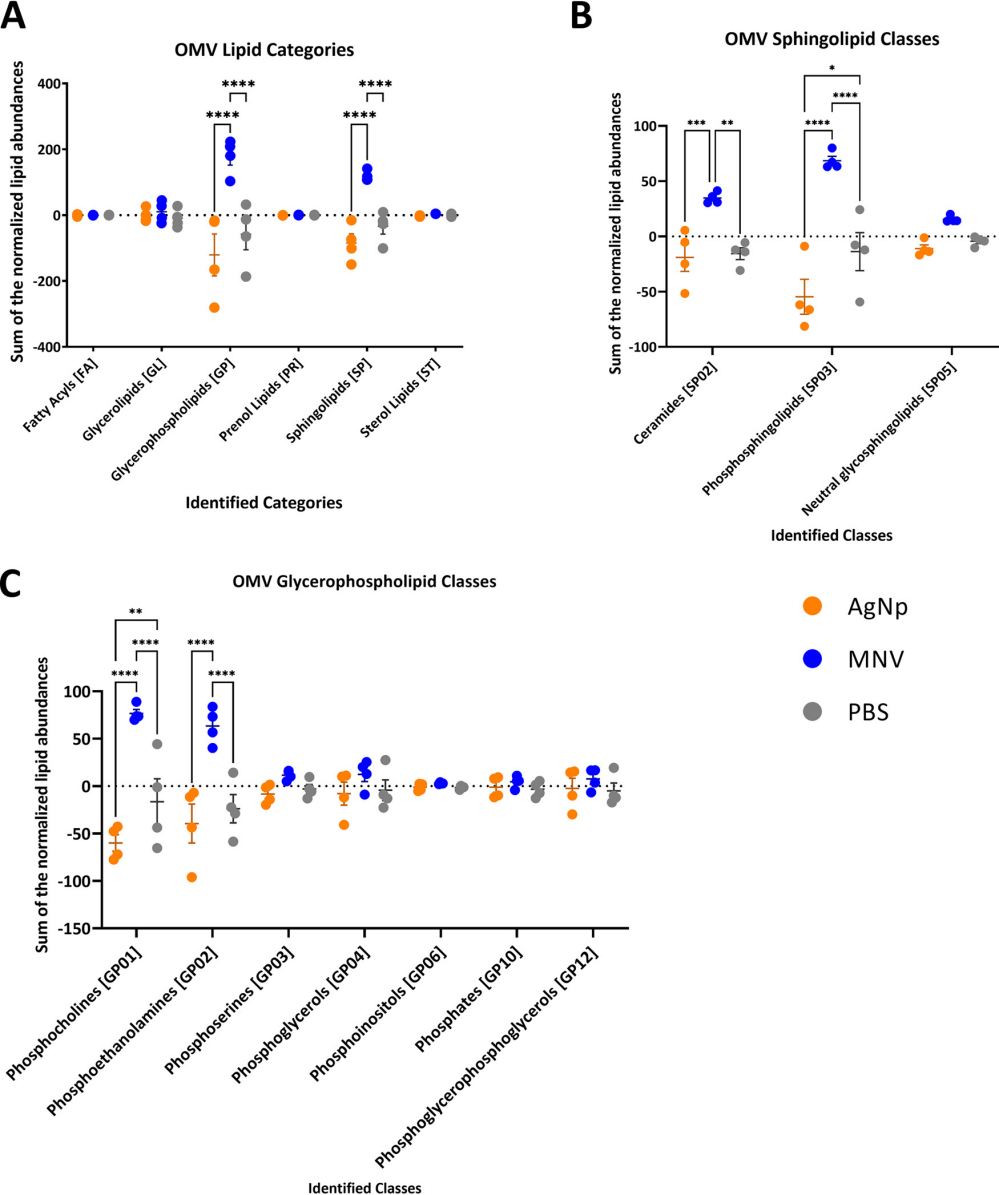
Summed abundances of the lipids in the bEVs. After normalization by sum and centered log-ratio transformation, the sum of the lipids was calculated for the (A) lipid categories. Further graphs show the sum of the classes for the (B) sphingolipid category and the (C) glycerophospholipid category. LipidMaps nomenclature tools were used to classify the identified lipids into categories and classes. The PBS condition samples are gray, MNV is in blue, and AgNp is in orange. Two-way ANOVA using Tukey’s multiple-comparison test was used to investigate statistical significance. Asterisks represent adjusted *P* values: *, *P* ≤ 0.05; **, *P* ≤ 0.01; ***, *P* ≤ 0.001; ****, *P* ≤ 0.0001. *n* = 4.

LipidMaps was used to assign lipid categories and classes to the identified lipids and illustrates that lipids belonging to the sphingolipid (SP) and glycerophospholipid (GP) categories predominate the differentially abundant lipids upon MNV interaction with bacteria (30) (Fig. 2C). Within these categories, ceramides, neutral glycosphingolipids, phosphosphingolipids, glycerophosphocholines, and glycerophosphoethanolamines are differentially abundant. To see how these categories and classes shaped the distribution of the lipids as a whole, rather than looking at only the differential lipids, a two-way ANOVA was used to analyze the total sum of the center-log transformed lipid abundances, first by category (Fig. 3A) and then by sphingolipid class and glycerophospholipid class (Fig. 3B and C). Multiple comparisons testing was used to compare the different conditions and showed a statistically significant increase in GP and SP categories for MNV compared with both PBS and AgNp controls (Fig. 3A). Within the classes of the SP category, there is increased abundance of ceramides and phosphosphingolipids in the MNV condition compared to either PBS or AgNp. In the GP classes, MNV led to a statistically significant increase in the glycerophosphocholine and glycerophosphoethanolamine classes. This marked change in the lipid profile of the OMVs in the presence of MNV could be indicative of increased vesiculation due to lipid accumulation resulting from membrane instability and/or could indicate selective packaging of lipids into OMVs under the condition of MNV-induced stress.

### Murine norovirus interaction does not result in a distinct lipidome of the bacterial outer membrane.

**FIG 4:**
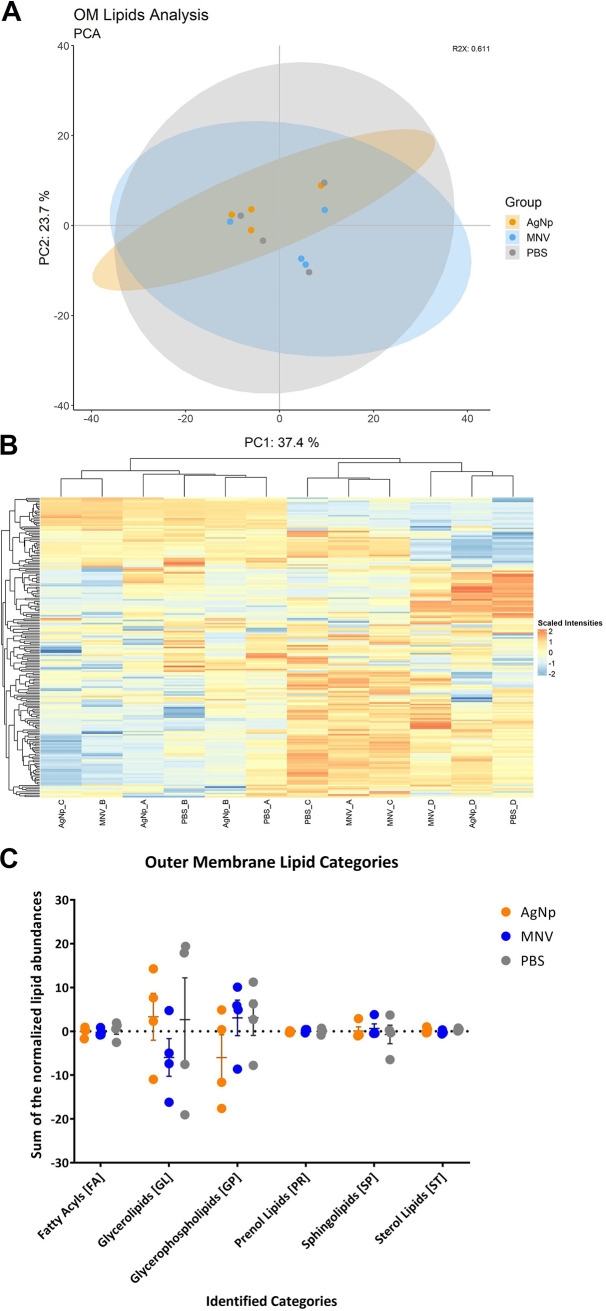
Lipidomic analysis of E. cloacae outer membranes. (A) Principal-component analysis (PCA) plot showing the lack of global difference in lipid composition of E. cloacae OMs under MNV, AgNp, and PBS conditions. Cultures of E. cloacae were incubated with MNV-1, PBS, or AgNp with PBS and AgNp serving as control conditions. (B) Heatmap showing the normalized and clr-transformed lipidomics results for E. cloacae OMs. (C) Summed abundances of the lipids in the bEVs. After normalization by sum and centered log-ratio transformation, the sum of the lipids was calculated for the lipid categories. The PBS condition samples are gray, MNV is in blue, and AgNp is in orange. *n* = 4.

Untargeted lipidomics was also performed on bacterial outer membrane (OM) extracts of the bacterial cultures from which the OMVs were collected, to determine if changes in OMV lipid content correspond to changes in the lipid content of the OM when MNV is present. The same four biological replicates of E. cloacae incubated with either PBS, 45-nm AgNp, or MNV were used for OM analysis via mass spectrometry and LipidMatch software to identify lipid changes. There was a combined total of 189 features identified across all sample types. A PCA plot of the lipid content in OM samples indicated a lack of separation between the MNV condition and the two control conditions (Fig. 4A). While in the OMV PCA plot the MNV samples are distinct from the PBS and AgNp samples (Fig. 2A), the OM lipidome shows all three conditions to be similar. This lack of significant differences can also be seen in a heatmap showing all the identified lipids, where no distinct clustering is observed (Fig. 4B). LipidR was again used to test for differentially abundant lipids using a log_2_-fold change cutoff of ±1 and an adjusted *P* value cutoff of <0.05. For the OM samples, there were no lipids found to be significantly differentially abundant between the MNV, PBS, and AgNp conditions (Table S2). The lack of significant differences between the conditions can be further seen in the total sum of the center log-transformed lipid abundances by category (Fig. 4C). Where GP and SP were significantly different for MNV in the OMV analyses, none of the lipid categories were significantly different in OM analyses between MNV, PBS, and AgNp when analyzed by two-way ANOVA. Together, these results indicate that while interactions with MNV lead to increased OMV production (11) and changes in OMV protein (11) and lipid content (Fig. 2 and 3), the virus does not induce similar changes in the parental bacterium OM.

### Murine norovirus interaction results in a limited amount of uniquely packaged bEV metabolomic content.

**FIG 5:**
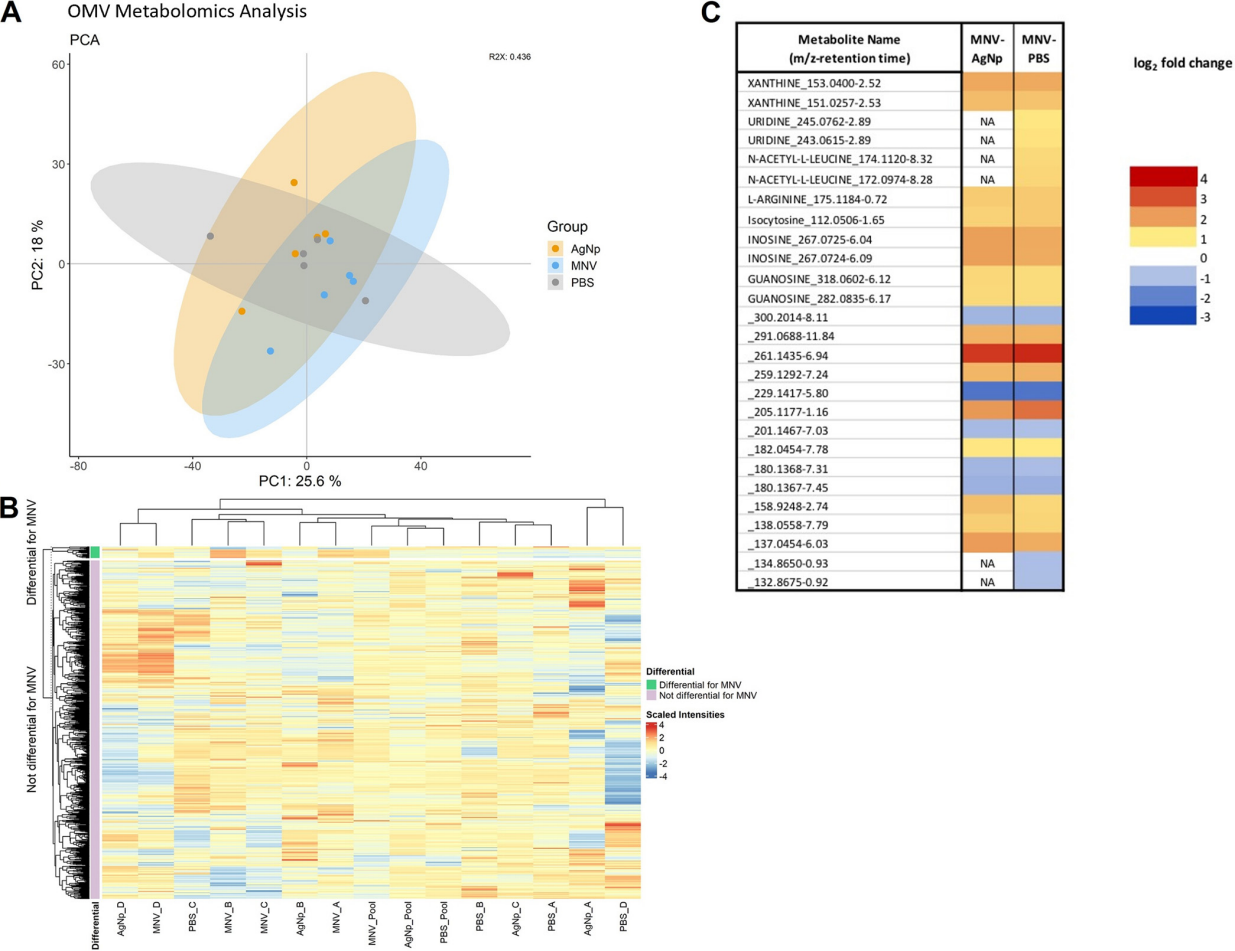
Metabolomic analysis of bEVs produced by Enterobacter cloacae. (A) Principal-component analysis (PCA) plot showing the metabolite composition of E. cloacae bEVs under MNV, AgNp, and PBS conditions. Cultures of E. cloacae were incubated with MNV-1, PBS, or AgNp with PBS and AgNp serving as control conditions. The PBS condition samples are gray, MNV is in blue, and AgNp is in orange. *n* = 4. (B) Heatmap showing the normalized and clr-transformed metabolomics results. The *y* axis coloration highlights metabolites found to be differentially abundant in the MNV condition in green, while the lilac coloration shows the metabolites not found to be statistically different between the conditions. (C) Heatmap showing metabolites that are differentially expressed between MNV and the AgNp and PBS control conditions. Color gradient represents the log_2_-fold change values of the statistically significant metabolites, all of which had an FDR of <0.05. NA indicates that particular comparison was not statistically significant. Identified metabolites from the internal standards are named; the rest are designated by *m/z* value and retention time.

The same E. cloacae OMVs and bacterial cell samples used for lipidomic analysis were also examined for their metabolomic content. The untargeted metabolomics include analysis of individual samples and pooled samples for each condition, and metabolites were identified with in-house standards. After filtering, a total of 823 metabolites were present in OMVs across both the negative and positive ion modes; however, only 159 of them were able to be identified with the standards used. Multivariant analysis was done using a PCA, which showed a lack of global separation between the different conditions, with all MNV, PBS, and AgNp samples overlapping each other (Fig. 5A). The heatmap provides further visualization of the lack of distinct clustering of the samples, although a small percentage (3.3%) of the identified metabolites were seen to be differential for the MNV condition compared to the controls (Fig. 5B). Comparing MNV versus AgNp resulted in the identification of 21 differentially abundant metabolites (log_2_-fold change ±1 and adjusted *P* values <0.05), while there were 27 metabolites found for the MNV versus PBS comparison. Of these, all 21 metabolites for MNV versus AgNp were also found in the MNV versus PBS condition, with similar trends of increased or decreased abundance for all of them (Fig. 5C, Table S3). Twelve of the 27 differential metabolites were able to be identified with the in-house standards. These include xanthine, uridine, n-acetyl-leucine, l-arginine, isocytosine, inosine, and guanosine, all of which were increased in abundance in MNV versus PBS or AgNp. No differentially abundant metabolites were seen when comparing the two control conditions to each other. While the percentage is low, the presence of differentially abundant metabolites in the MNV condition compared to the controls further suggests a level of preferential packaging of content into OMVs.

### Murine norovirus interaction does not significantly affect bacterial metabolomic content.

**FIG 6:**
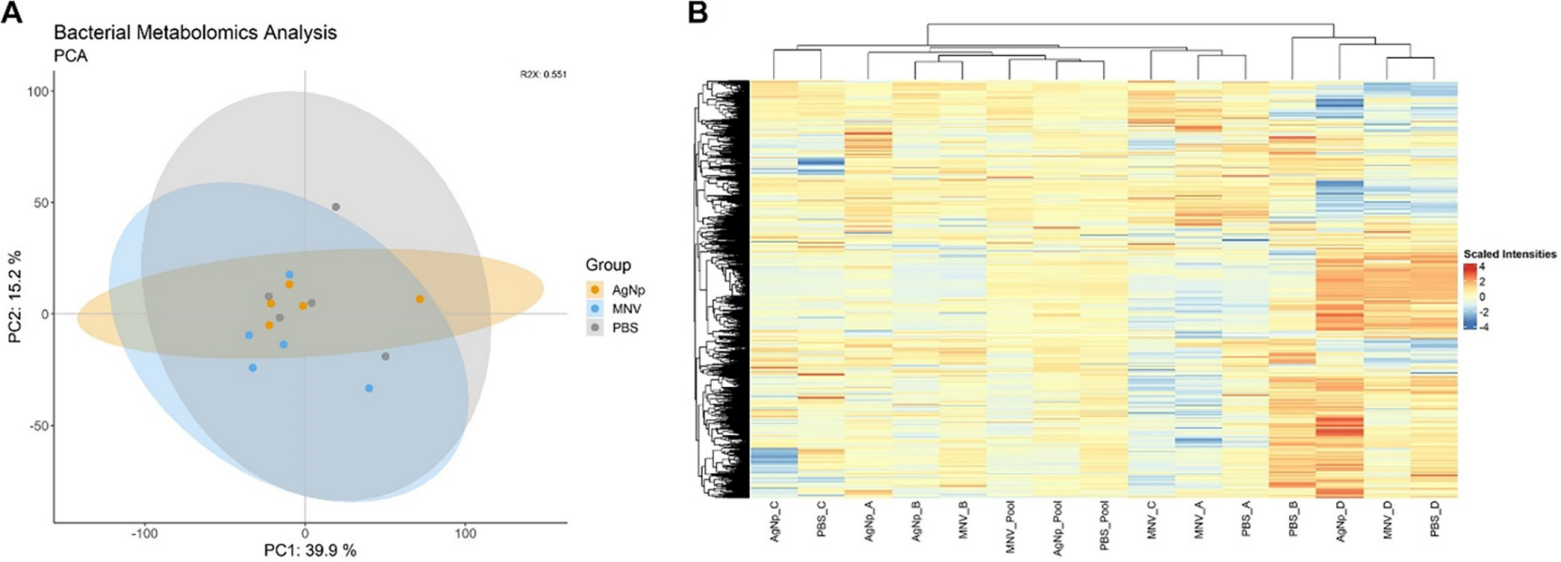
Metabolomic analysis of E. cloacae cell lysates. (A) Principal-component analysis (PCA) plot showing a lack of global differences in metabolite composition of E. cloacae bEVs under MNV, AgNp, and PBS conditions. Cultures of E. cloacae were incubated with MNV-1, PBS, or AgNp with PBS and AgNp serving as control conditions. The PBS condition samples are gray, MNV is in blue, and AgNp is in orange. *n* = 4. (B) Heatmap showing the normalized and clr-transformed metabolomics results.

Untargeted metabolomic analysis was also performed on the whole bacterial cell lysates for E. cloacae incubated with either PBS, AgNp, or MNV. Like the metabolomics of OMVs, pooled samples for each condition were also included, and metabolite identities were confirmed with in-house standards. As expected, a considerably larger number of metabolites were identified in the bacterial cells than the OMVs, with a total of 2,499 metabolites seen in the negative and positive ion modes, with 379 that match in-house standards. Both the PCA plot and the heatmap for the bacterial metabolomics show a distinct lack of separation of the MNV, PBS, and AgNp conditions (Fig. 6, Table S4). Further analysis revealed that there were no differentially abundant metabolites for MNV versus the control conditions, nor for AgNp versus PBS, indicating that the interaction of MNV with E. cloacae does not substantially alter the metabolites in the bacterial cell. This observation is in contrast to the results for the OMV metabolomics, which, while also not showing clear separation in the PCA plot, did have a small percentage of metabolites that were differentially abundant in the MNV condition (Fig. 5).

## DISCUSSION

Understanding the mechanisms of membrane vesicle biogenesis and the packaging of content that could result in changes in bacterial communication with other cells is an ongoing area of research. In this study, we used lipidomics and metabolomics to further analyze the content of OMVs produced by the commensal bacterium E. cloacae upon bacterial exposure to the eukaryotic pathogen murine norovirus. We have previously shown that human norovirus particles and MNV induce expression of stress response genes by E. cloacae and concomitantly induce hypervesiculation and changes in OMV protein and DNA content (11). It has been previously suggested that E. cloacae is capable of forming vesicles through explosive cell lysis due to the abundance of cytoplasmic proteins and genomic DNA found in these vesicles (27); therefore, the decrease in cytoplasmic proteins and DNA indicated a shift away from explosive cell lysis and possibly toward the membrane blebbing mechanism of vesicle biogenesis (1, 7). To further explore this potential shift in vesicle biogenesis route, we used a cell viability stain determine if there were changes in the number of cells with damaged membranes in the presence of MNV, as damaged membranes could indicate explosive cell lysis. The cell viability assay results show an increase in the ratio of SYTO 9 (cells with intact membranes) to propidium iodide (cells with damaged membranes) in the MNV condition compared to the PBS and AgNp conditions (Fig. 1). This suggests fewer cells with damaged membranes in the MNV condition, supporting our previous findings indicating that interaction with MNV prompted a shift away from the explosive cell lysis mechanism of vesicle biogenesis (11). Furthermore, it has been hypothesized that the changes in phospholipids present in the membrane vesicles compared to the bacterial outer membrane are driven by the need for a higher curvature in the vesicles, which would be expected during hypervesiculation and membrane blebbing (4, 31). Lipids such as phosphatidylglycerol, phosphatidylethanolamine, and cardiolipin are commonly found in bacterial vesicles (32 to 35) and also found in E. cloacae OMVs. As mentioned above, MNV induces E. cloacae to increase OMV production, and lipid accumulation is also observed in these OMVs compared to those produced by control bacteria, indicating that MNV interaction not only leads to hypervesiculation but also influences the accumulation of lipids in the OMVs, again supporting an increase in membrane blebbing biogenesis.

Lipidomic analysis of E. cloacae OMVs also revealed that a number of glycerophospholipid species, including phosphatidylethanolamine, were also differentially abundant in the MNV condition compared to the controls for the OMVs (Fig. 2C), and a general increase in glycerophospholipids, particularly phosphocholines and phosphoethanolamines, were also observed under this condition (Fig. 3). Work by Roier et al. showed that a shift in the lipidome of Haemophilus influenzae was seen in hypervesiculation mutants where OMVs were enriched in glycerophospholipids, particularly phosphatidylethanolamine (PE) (35). Therefore, our findings in E. cloacae suggest that a shift in the lipid architecture of OMVs created in the presence of MNV may be driven by an accumulation of phospholipids resulting in increased blebbing, similar to the findings by Roier et al. (35). Moreover, other studies have noted that the deletion of genes involved in envelope stability and lipid synthesis affected the size distribution and content of OMVs (36). However, additional experiments using gene knockout mutants would be needed to determine if increased phospholipid accumulation is a pathway used by E. cloacae to increase OMV production.

Besides glycerophospholipids, a number of lipid species differentially abundant after MNV interaction are classified as sphingolipids. It was long thought that these lipid species were not present in bacteria, but research has shown that the ability to synthesize these lipids can be found in multiple genera of Gram-negative bacteria (37 to 39). Consistent with our results in E. cloacae, it was recently shown that sphingolipids could be incorporated in vesicles produced by Bacteroides thetaiotaomicron (40). While the specific role of bacterial sphingolipids is still being investigated, sphingolipids produced from host cells are involved in inflammatory responses and can mediate inflammatory signaling (41). Our group has previously demonstrated that OMVs from both B. thetaiotaomicron and E. cloacae induce inflammatory responses in macrophages and subsequently control norovirus infection in these cells (42). Interestingly, OMV production in the intestinal tract increases during MNV infection (11); therefore, the increased presence of sphingolipids in OMVs that were produced in the presence of MNV may be a primary vesicular component regulating host immune responses to infection.

Approximately a third of the identified lipids were differentially abundant in the vesicles formed in the presence of MNV compared to the controls. Meanwhile, the identified lipids in the E. cloacae OMs align with those seen in previous works (16, 20, 34), but there were no statistically significant changes in the lipid composition of the OM across the different conditions (Fig. 3). Previous literature has shown that during the membrane blebbing biogenesis route the lipid profile of the OMVs is often similar to that of the outer membrane, but the abundance of lipid species differs between the two (40, 43, 44). Our data normalize the vesicle lipidomics results and the bacterial OM lipidomics results separately, preventing direct comparison of the numbers against each other. However, it can clearly be seen in the vesicles that there is a predominance of glycerophospholipids followed by the sphingolipids and then glycerolipids in both the abundance and the number of identified lipids for the respective categories (Fig. 2A, Table S5). In contrast, the OM is most abundant in glycerolipids followed by glycerophospholipids and sphingolipids, while the number of identified lipids for each category shows glycerophospholipids as the most identified followed by glycerolipids (Fig. 3C, Table S5). Thus, we do see a differential abundance of lipid species between vesicles and the parental OM, which may indicate that membrane blebbing is the mechanism of vesicle biogenesis. However, since differences in the lipid content in response to MNV is only observed in the vesicles and not in the cellular OM, this may also indicate that MNV induces selective packaging of lipids into vesicle cargo, such as for the targeted release of lipids in the vesicles to maintain the fluidity of the outer membrane (43). An additional explanation for the differences seen in the vesicles but not in the OM is that the MNV condition has a different ratio of OMVs to OIMVs compared to the control conditions, and that these different vesicle formation routes alter the lipid profiles enough to be seen in the differential abundance of the lipidomics results. Further experimentation using bacterial mutants is under way to determine if MNV-induced changes are due to vesicle formation versus selective packaging.

Literature dealing with metabolomics of OMVs is limited, with even fewer studies looking into how they may relate to the biogenesis of the vesicles (4, 45). Studies of OMVs produced by *Bacteroides* spp. have used metabolomics to show that there is selective packaging of metabolites into OMVs, often involving packaging of metabolites designed to improve fitness in specific environmental niches (46, 47). Analysis of metabolites can be further hindered by the fact that it is difficult to confirm the identity of the metabolites in the absence of a standard. In our study, only 44% (12 of 27) of the metabolites shown to be differentially abundant in the MNV condition versus PBS or AgNp were identified (Fig. 4C). Nearly half of the differentially abundant metabolites identified in MNV OMVs are involved in purine metabolism (xanthine, inosine, and guanosine), while l-arginine is involved in the metabolism of arginine, proline, aspartate, glycine, and serine. These metabolites were seen to be increased in the MNV OMVs compared to the PBS or AgNp controls. These results indicate a possible preferential packaging of metabolites into OMVs when MNV is present. In addition, the presence of these metabolites may also be indicative of changes in gene expression leading to changes in membrane stability. l-arginine is a primary amino acid used to synthesize polyamines (48). The presence of polyamines in cellular structures increases membrane stability, and reduction in polyamine synthesis is linked to increased membrane fluidity (49 to 51). Our previous analysis of E. cloacae gene expression in the presence of MNV showed that expression of genes used to convert l-arginine to the polyamine putrescine (*speA* and *speB*) (48) were significantly downregulated in the presence of norovirus. Downregulation of these genes would likely lead to l-arginine accumulation, a reduction in polyamine concentration, and reduced membrane stability. This finding would be consistent with our lipidomics results showing the increased presence of phospholipids which lessen membrane rigidity to allow for increased membrane curvature and vesicle blebbing.

The metabolomic analysis of the bacterial cells produced results similar to what was observed with the bacterial OM lipidomics, where there was no distinction of metabolite content among the samples. No metabolites in the bacterial lysates were found to be differentially abundant, nor was there distinct clustering of the sample conditions (Fig. 5) similar to what was observed for the OMVs. This difference between what is seen in the OMVs compared to the bacterial cells may indicate that any shift in metabolites caused by interaction with MNV is being packaged into the OMVs and not remaining in the parental cell.

In conclusion, the lipidomics analysis of OMVs show a distinct lipidome in OMVs formed in the presence of MNV compared to OMVs from PBS and AgNp conditions; however, there does not appear to be a significant difference in the lipidome of the bacterial outer membrane. In addition, an increase in phospholipids in the MNV condition supports the budding route of OMV biogenesis through phospholipid accumulation in the outer membrane (2, 35, 45). The metabolomics results for MNV OMV suggest preferential packaging of specific metabolites, but the same distinction of MNV from the controls is not seen in the bacterial cell metabolomics. The differences in lipids and metabolites detected in OMVs but not the parental bacteria in the presence of MNV may indicate that any changes to lipids or metabolites by interaction with MNV results in the packaging of those lipids or metabolites into OMVs for removal from the cell. Finally, the detection of fewer bacterial cells with damaged membranes upon the presence of MNV, further supports a shift of OMV biogenesis upon the virus bindings, from partial formation by explosive cell lysis to a blebbing mechanism.

## MATERIALS AND METHODS

### Bacterial strains and growth conditions.

Enterobacter cloacae (ATCC no. 13047) was cultivated in Luria-Bertani (LB) broth or agar consisting of 1% tryptone, 1% sodium chloride, and 0.5% yeast extract, plus 1.5% agar for the agar plates. Isolates were grown under aerobic conditions at 37°C with constant shaking (220 rpm). Ingredients to make LB were purchased from Thermo Fisher Scientific. Glycerol stocks of the bacteria were stored at −80°C in LB with 50% (vol/vol) glycerol. Representative growth curves can be seen in Fig. S1.

### Murine norovirus.

Recombinant murine norovirus-1 (MNV-1) was generated using plasmid pSPMNV-1.CW3 (provided by Stephanie Karst, University of Florida) as previously described (52). Briefly, 5 μg of pSPMNV-1.CW3 was used to transfect 293T cells. After 24 h, the cells were harvested and lysed, and the supernatant was collected for centrifugation. The titer of the supernatant was determined by a TCID_50_ assay. The clarified supernatant was then used to infect RAW264.7 cells at an MOI (multiplicity of infection) of 0.05 and harvested 36 to 48 h postinfection. Freeze-thaw cycles were used to lyse the cells before the supernatant was subjected to ultracentrifugation through a 25% sucrose cushion. The resulting pellet containing the virus was resuspended in dPBS before aliquoting into 20-μL amounts. The final viral titer was determined using a TCID_50_ assay. The resulting MNV stocks were stored at −80°C until use.

### Viral attachment assay.

Viral attachment assays were performed as described previously (18). Bacteria were grown in 120 mL of LB until they reached the stationary phase. The bacterial pellet was washed twice with 1× PBS and concentrated into 5 mL of PBS. The bacterial cell count was adjusted with PBS to a final concentration of 10^8^ cells/mL based on the OD_600_ reading corresponding to previously done growth curves, and serial dilution plating was used to verify the concentration. Cells were inoculated with either MNV (0.1 MOI), silver nanoparticle (AgNp; 0.1 μg/mL) or PBS. PBS and AgNp were used as controls, where AgNp served as an abiotic particle control of similar physical size to noroviruses but lacking viral antigens, and PBS was the diluent for both MNV and AgNp (11). The concentration for AgNp was chosen because it approximated the number of virus particles in our MNV inoculum, as was determined in our previous publication (11). The concentration of AgNp used is below the amount needed for the silver to affect bacterial growth, as evidenced by Fig. S1B. The mixtures were incubated for 1 h at 37°C with constant mixing.

### Bacterial membrane vesicle generation and isolation.

Generation of OMVs from E. cloacae cultures was performed as previously described (11). Following the 1 h of incubation step in the virus-bacteria attachment assays, the virus-bacteria cultures were inoculated into 60 mL of fresh LB and grown at 37°C for 12 h. Cultures were then centrifuged at 2,000 × *g* for 20 min at 4°C to pellet out bacterial cells. The supernatants of the cultures were then ultracentrifuged at 25,000 × *g* for 20 min at 4°C. The resulting supernatant was next passed through a 0.22-μm filter into new ultracentrifuge tubes and ultracentrifuged at 150,000 × *g* for 2 h at 4°C. OMV pellets were resuspended in dPBS and ultracentrifuged once more at 150,000 × *g* for 2 h at 4°C before a final resuspension in 500 μL of 1× protease inhibitor cocktail (Thermo Fisher no. A32955) made with dPBS. OMVs were stored at 4°C before being sent to the Southeast Center for Integrated Metabolomics (SECIM) for extraction within 1 week. To ensure removal of parental bacteria from vesicle extracts, 10 μL spots of the isolated OMVs were plated on LB agar.

### Cell viability assay.

Cell viability assays were carried out using the LIVE/DEAD BacLight Bacterial Viability kit (Thermo Fisher) according to the manufacturer’s instructions. Similar to the procedure for bacterial extracellular vesicle generation, after the 1 h incubation step in the virus–bacteria attachment assays, the virus-bacteria cultures were inoculated into 30 mL of fresh LB and grown at 37°C for 12 h. The cultures were spun down and the supernatant removed before the pellet was resuspended, and all conditions were diluted to an OD_600_ of 0.22. A subset of E. cloacae from the PBS condition was killed in 70% ethanol and used as a control for dead/damaged cells.

### Outer membrane extraction from E. cloacae.

The outer membrane for the lipidomics experiment was extracted from the bacterial pellet formed after the first spin mentioned above in the Bacterial Membrane Vesicle Generation and Isolation methodology. The pellets were washed twice with HEPES buffer and centrifuged for 10 min at 2,000 × *g* at 4°C. Pellets were resuspended in a HEPES buffer and protease inhibitor cocktail before passing through a French press at 1,000 lb/in^2^ five times. The lysed cell suspension was spun at 10,000 × *g* for 10 min at 4°C to remove cellular debris. The supernatant was poured into ultracentrifuge tubes for a 1-h spin at 4°C at 100,000 × *g*. The pellet was resuspended in HEPES buffer before another ultracentrifuge spin with the same settings. The resulting pellet was resuspended in 1% (wt/vol) N-lauroylsarcosine diluted in HEPES buffer. After a 30-minute incubation at 37°C, the sample was spun again for 1 h at 4°C at 100,000 × *g*. Another wash step with HEPES buffer and another ultracentrifuge spin followed before a final resuspension in 500 μL of HEPES buffer. Samples were stored at 4°C before being sent off for lipidome analysis.

### Lipidomics.

Lipidomics were performed on OMVs and bacterial outer membranes. Lipid extraction using the Folch extraction method through to the creation of a peak intensity table was performed by the Southeast Center for Integrated Metabolomics (SECIM) at the University of Florida. For the Folch extraction method, 100 μL of OMV or 400 μg/mL of bacterial outer membrane extract was used. The organic layer was dried and reconstituted at 50 μL with an injection standard solution in 2-propanol. Global lipidomics profiling was performed on a Thermo Q-Exactive Orbitrap mass spectrometer with Dionex UHPLC and autosampler. All samples were analyzed in positive and negative heated electrospray ionization with a mass resolution of 35,000 at *m/z* 200 as separate injections. Separation was achieved on an Acquity BEH C18 1.7-μm, 100 × 2.1 mm column with mobile phase A as 60:40 acetonitrile:10 mM Ammonium formate with 0.1% formic acid in water and mobile phase B as 90:8:2 2-propanol:acetonitrile:10 mM ammonium formate with 0.1% formic acid in water. The flow rate was 500 μL/min with a column temperature of 50°C. Five microliters was injected for negative ions and 3 μL for positive ions. Data from positive and negative ion modes were separately analyzed using LipidMatch software. First, all MS2 raw files were converted to .ms2 and MS raw files to. MzXML using MSConvert. A peak list was generated after running MzMine on all MzXML files. An input folder that included all .ms2 files and the peak list were used to run LipidMatch to identify features.

### Metabolomics.

Metabolomics were performed on OMVs and bacterial cells taken from the bacterial pellet formed after the first spin mentioned above in the Bacterial Membrane Vesicle Generation and Isolation methodology. Metabolomics procedures from the extraction to the creation of a peak intensity table were carried out by the Southeast Center for Integrated Metabolomics (SECIM) at the University of Florida. After extraction, the aqueous layer was dried and reconstituted at 50 μL with injection standard solution in water with 0.1% formic acid for liquid chromatography-mass spectrometry (LC-MS) analysis. Global metabolomics profiling was performed on a Thermo Q-Exactive Oribtrap mass spectrometer with Dionex UHPLC and autosampler. All samples were analyzed in positive and negative heated electrospray ionization with a mass resolution of 35,000 at *m/z* 200 as separate injections. Separation was achieved on an ACE 18-pfp 100 × 2.1 mm, 2-μm column with mobile phase A as 0.1% formic acid in water and mobile phase B as acetonitrile. The polar embedded stationary phase provides comprehensive coverage but has some limitations in covering very polar species. The flow rate was 350 μL/min with a column temperature of 25°C. Four microliters of the sample was injected for negative ions and 2 μL for positive ions. Data from positive and negative ion modes were separately analyzed using MZmine software to identify features, deisotope, align features, and perform gap filling to fill in any features that may have been missed in the first alignment algorithm. All adducts and complexes were identified and removed from the data set. The data were searched against the SECIM internal retention time metabolite library to identify known metabolites.

### Data analysis and statistics.

Following the delivery of the peak tables for the lipidomics and metabolomics data, the positive and negative modes were combined before being uploaded to the MetaboAnalyst (53) website, where features with >50% missing values were removed, and the remaining missing values were replaced with the estimated limit of detection (1/5 of the minimum positive value of each variable) before the data were filtered and normalized by sum. The data were then imported into RStudio (54, 55), where the R package “compositions” (56) was used to transform the data with centered log-ratio (clr) coefficients. This approach is used for compositional data as it combines scaling the data based on geometric means and a logarithm transformation to normal distribution. The principal-component analysis plots and differential abundance analysis were run using the “lipidr” R package (29), while the heatmap was generated using the “complex heatmap” R package (57). One-way ANOVA was used to analyze the cell viability assay results with Tukey’s multiple-comparison test. Statistical significance of the summed abundances for the lipidomics categories and classes was measured using two-way ANOVA with Tukey’s multiple-comparison test. All biological replicates have at least three replicates, and where possible, at least three technical replicates were used. Graphs and plots were made using either GraphPad Prism (version 8.0.0 for Windows, GraphPad Software, San Diego, California USA, www.graphpad.com) or R Studio (version 1.3.1093 with R version 4.0.2) (54, 55).
