# Supplementary material for: Murine Norovirus Interaction with Enterobacter cloacae Leads to Changes in Membrane Stability and Packaging of Lipid and Metabolite Vesicle Content
Source: Microbiol Spectr. 2023 Mar 21;11(2):e04691-22. doi: 10.1128/spectrum.04691-22 (PMC10100888; doi:10.1128/spectrum.04691-22)
Supplement: Supplemental file 2 — Supplemental material. Download spectrum.04691-22-s0002.pdf, PDF file, 1.0 MB [file spectrum.04691-22-s0002.pdf]

SUPPLEMENTAL MATERIALS AND METHODS

Bacterial Growth Curves

Enterobacter cloacae growth curves were created by inoculating a single isolated colony from a fresh agar plate (less than one week old) into 5 mL of LB broth in a conical tube. The cultures were then incubated overnight at 37 °C with aerobic shaking at 200 rpm. After overnight incubation, 25 mL of LB broth was inoculated 1:100 with the overnight culture. At the specified time intervals, 1 mL of culture was removed and used to read absorbance (600nm) and for serial dilution and plating onto LB agar. Agar plates were incubated overnight at 37 °C. After incubation, colonies on plates were counted and the CFU/mL was calculated for each time point.

To determine if either silver nanoparticles (AgNp) or murine norovirus (MNV) affected the growth of *E. cloacae*, additional growth curves were created using a 96-well plate with 200 µL of LB broth in the wells. Overnight cultures of *E. cloacae* were adjusted with PBS to a final concentration of 10^8 cells in 1 mL based on the OD_{600} reading corresponding to previously done growth curves. Cells were then inoculated with either MNV (0.1 MOI) or silver nanoparticle (AgNp; 0.1 µg/mL). After thorough mixing, 5 µL of each biological replicate was inoculated into 195 µL of LB broth in each test well. The 96-well plate was then incubated at 37°C, with absorbance readings using a spectrophotometer being taken at the specified timepoints.

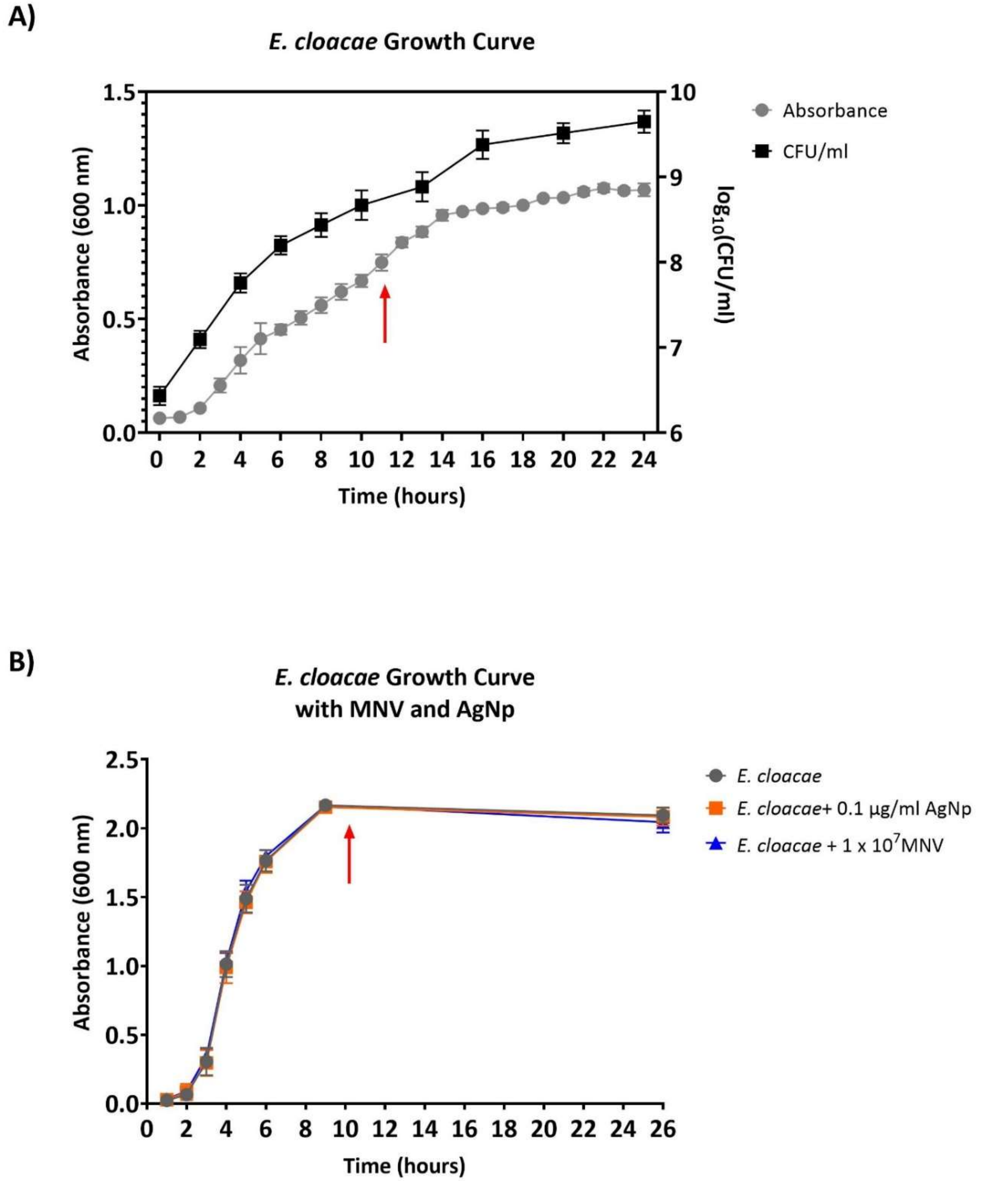

Figure S1. Representative growth curves of *Enterobacter cloacae.* Red arrows mark the 12 hour point where vesicles are collected. **A)** *E. cloacae* growth curve showing optical density as measured by absorbance at 600 nm on the left axis and in grey. Colony forming units are displayed on the right y-axis and in black for certain time points. N = 3. **B)** A microplate reader was used to measure absorbance of *E. cloacae* with silver nanoparticles (AgNp) or murine norovirus (MNV). N = 3.
